# Optimized molecular resolution of cross-contamination alerts in clinical mycobacteriology laboratories

**DOI:** 10.1186/1471-2180-8-30

**Published:** 2008-02-14

**Authors:** Ana Martín, Marta Herranz, Miguel Martínez Lirola, Rosa Fernández Fernández, INDAL-TB group, Emilio Bouza, Darío García de Viedma

**Affiliations:** 1 Servicio de Microbiología Clínica y Enfermedades Infecciosas. Hospital Gregorio Marañón, Universidad Complutense, Madrid, CIBER-Enfermedades Respiratorias CIBERES, Spain; 2 Servicio de Microbiología, Complejo Hospitalario Torrecárdenas, Almería, Spain; 3 Unidad de Tuberculosis del Distrito Poniente, Almería, Spain

## Abstract

**Background:**

The phenomenon of misdiagnosing tuberculosis (TB) by laboratory cross-contamination when culturing *Mycobacterium tuberculosis *(MTB) has been widely reported and it has an obvious clinical, therapeutic and social impact. The final confirmation of a cross-contamination event requires the molecular identification of the same MTB strain cultured from both the potential source of the contamination and from the false-positive candidate. The molecular tool usually applied in this context is IS6110-RFLP which takes a long time to provide an answer, usually longer than is acceptable for microbiologists and clinicians to make decisions. Our purpose in this study is to evaluate a novel PCR-based method, MIRU-VNTR as an alternative to assure a rapid and optimized analysis of cross-contamination alerts.

**Results:**

MIRU-VNTR was prospectively compared with IS6110-RFLP for clarifying 19 alerts of false positivity from other laboratories. MIRU-VNTR highly correlated with IS6110-RFLP, reduced the response time by 27 days and clarified six alerts unresolved by RFLP. Additionally, MIRU-VNTR revealed complex situations such as contamination events involving polyclonal isolates and a false-positive case due to the simultaneous cross-contamination from two independent sources.

**Conclusion:**

Unlike standard RFLP-based genotyping, MIRU-VNTR i) could help reduce the impact of a false positive diagnosis of TB, ii) increased the number of events that could be solved and iii) revealed the complexity of some cross-contamination events that could not be dissected by IS6110-RFLP.

## Background

The false diagnosis of tuberculosis (TB) due to laboratory cross-contamination is a well-known phenomenon and has been reported to occur in 0.1–3% of cases [1-5]. It has an obvious epidemiological, clinical and therapeutical impact -each misdiagnosis of tuberculosis due to laboratory cross-contamination has been estimated to cost on average 10,872 dollars [6]. We recently presented data indicating that laboratory cross-contamination events are more frequent than expected [7] and that these alerts require a faster clarification. False positivity due to laboratory cross-contamination is suspected when i) *Mycobacterium tuberculosis *(MTB) is cultured from only one of the serial specimens of a patient, ii) the bacterial yield in the culture is low and iii) the suspected sample has been processed together (or in a short period of time apart) with at least one other from a patient with a high bacterial load. The final confirmation of false positivity requires the application of molecular tools to prove that the MTB isolates from the co-processed specimens share identical genotypic patterns (after having ruled out epidemiological links between the cases involved). Unfortunately, the reference MTB genotyping method, IS6110-RFLP, requires well-grown cultures and takes a long time to provide an answer, usually longer than is acceptable for microbiologists and clinicians to make decisions.

A rapid PCR-based MTB genotyping tool, MIRU-VNTR (Mycobacterial-interspersed-repetitive-units-Variable-number-tandem-repeats) [8], has recently been developed and has proved useful in different epidemiological studies [9,10]. It could allow quicker resolution of cross-contamination alerts although studies evaluating the efficiency of MIRU-VNTR in this context in a prospective design are lacking, and only a few isolated examples of its potential in identifying false-positive cases have been reported [11]. Our purpose in this study is to evaluate MIRU-VNTR as an alternative to assure a rapid analysis of cross-contamination alerts in reference laboratories.

## Results and Discussion

We prospectively evaluated whether MIRU-VNTR could be an alternative to RFLP for the fast resolution of laboratory cross-contamination alerts in reference genotyping centres. Therefore, we applied both IS6110-RFLP and MIRU-VNTR in a pilot study to analyze all the alerts received from laboratories in Almería, Spain. We compared the response time and the correlation between the diagnosis of either true positivity or laboratory cross-contamination using both techniques. The response time was measured from the moment the culture was received until MIRUtype for all the 12 loci assayed, or an RFLPtype were obtained. We decided to accept only those results obtained within a reasonable time frame (below 90 days), because longer times were not considered useful for resolving the alerts.

**Figure 1:**
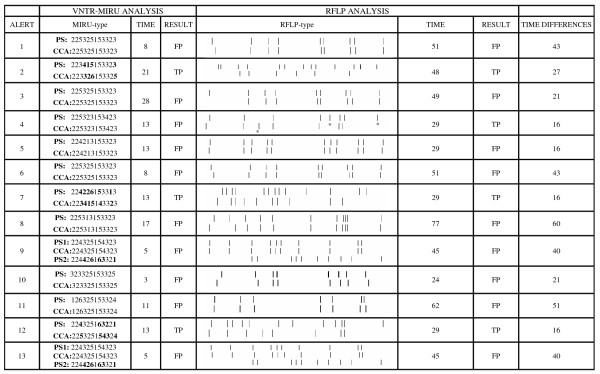
The numbers for each alert were assigned chronologically. For each alert the potential source/s (PS) and the cross-contamination alerts (CCA) are indicated. The MIRUtypes and RFLPtypes and the time (days) for the obtention of results, the result (TP:True positivity, FP: False positivity) after analyzing the MIRU-VNTR and RFLP fingerprints, and the differences between the time for solving the alerts by MIRU-VNTR and RFLP are shown. The asterisks in alert 4 indicate the differential bands between the RFLP patterns. The MIRU-VNTR alleles which are different for the isolates from an alert are highlighted in bold.

**Figure 2:**
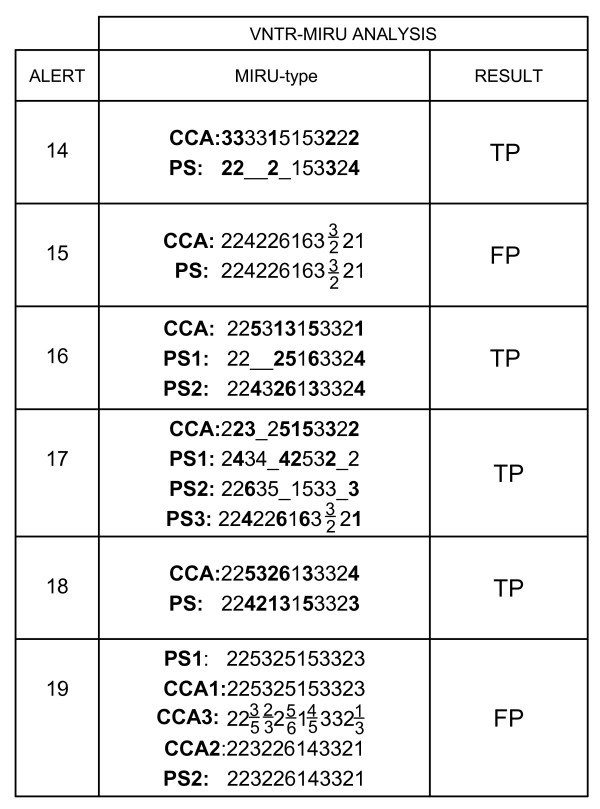
Analysis by MIRU-VNTR of the cross-contamination alerts that could not be solved by RFLP. The numbers for each alert were assigned chronologically. For each alert the potential source/s (PS) and the cross-contamination alerts (CCA) are indicated. The MIRUtypes and the results (TP:True positivity, FP: False positivity) after analyzing the MIRU-VNTR fingerprints are shown. The MIRU-VNTR alleles which are different for the isolates from an alert are highlighted in bold. The loci which appear underscored mean that no amplification product was obtained. The loci with two values indicate that two alleles were simultaneously detected for that locus.

Between November 2005 and February 2007, 19 cross-contamination alerts were received in our laboratory. If the potential sources (candidates of true-positive cases) and receptors (candidates of false-positive cases) in contamination events are taken together, a total of 48 cultures were involved (Figures 1 &2). In all cases MIRU-VNTR resolved the alert before RFLP. The response time range for MIRU-VNTR from the reception of the sample was 3–28 days (median: 13 days) whereas RFLP required 24–77 days (median 45 days) (Figure 1). This means that MIRU-VNTR reduced the standard response time by 16–60 days (median: 27 days), which could easily have been increased if priority had been given to performing MIRU-VNTR analysis. Instead, we preferred to perform this evaluation in the real situation of a genotyping laboratory with many other routines. The response time for MIRU-VNTR was longer than could be expected because the primary culture had to be sent to another reference laboratory to perform susceptibility assays, and this meant the reception in many cases of fresh subcultures that required preincubation before analysis. The application of capillary electrophoresis to analyze MIRU-VNTR products [8] had also reduced the response time. With regard to the correlation in the answers, RFLP detected false-positivity in eight of the thirteen events in which it was able to provide a response, indicating that the cross-contamination alerts were justified. MIRU-VNTR, even having applied the 12-loci set which is less discriminatory than the 15 or 24-loci sets [12,13], correlated in all cases except in one alert (alert 4) considered as true positivity by RFLP whereas MIRU-VNTR found identical genotypes for the involved cases. To try to clarify this discrepancy, MIRU-VNTR with the whole set of 24 loci was applied and, again, the involved cases shared identical MIRUtypes. A detailed analysis of the RFLPtypes indicated that most of the bands in alert 4 were shared and that differences could result from the acquisition of an additional *Pvu*II site responsible for the digestion of a long band into two smaller bands (Figure 1). One possible explanation for this potential discrepancy would be that the potential source case was infected by two IS6110 RFLP variants and only one was involved in the cross-contamination event. Consistent with this hypothesis, an independent specimen from the source case was genotyped and the same IS6110 profile as the one displayed in the contamination event was observed (data not shown).

As indicated above, RFLP could not offer an answer in six alerts, either because some of the cultures (in three alerts) did not lead to the bacterial yield required for RFLP or because the 90-day limit was exceeded. In all the cases that remained unsolved by RFLP, MIRU-VNTR provided a result and, in two of them, it identified a cross-contamination that would have gone undetected if only RFLP analysis had been available. Each one of these two cross-contamination events revealed only by MIRU-VNTR showed interesting features (Figure  2). The first one (alert 15) involved a polyclonal isolate, with two variants in one loci, similar to others previously reported [14]. The second (alert 19) was a complex situation in which two cross-contamination events occurred simultaneously. Each of two different sources (PS1 and PS2) contaminated a specimen from two independent patients (CCA1 and CCA2) and both (PS1 and PS2) were also involved in the simultaneous contamination of an additional specimen from another case (CCA3). This double contaminated case (CCA3) could be detected by MIRU-VNTR because its pattern was the combination of the MIRUtypes of the sources (PS1 and PS2), with two different alleles in three of the loci (Figure 2). In this alert, the 90-day limit was exceeded by RFLP and the profiles are not shown; however, the complex patterns of the isolates involved (14 bands and 8 bands) led to a 22-band pattern in the double-contaminated false-positive case that prevented identification of false-positivity by RFLP but not by MIRU-VNTR.

## Conclusion

Our data mean that MIRU-VNTR is more adequate than RFLP for analyzing cross-contamination alerts. It was faster than RFLP, the correlation with RFLP diagnosis was high and it succeded in resolving alerts even under circumstances that were not appropriate for the RFLP analysis requirements. A permanently suspicious attitude on the part of the clinical mycobacteriologist together with access to a fast resolution of cross-contamination alerts could enable more rapid management of suspected false-positive cases, because culture-results would only need to be retained for a short time before clarification. Unlike standard RFLP-based genotyping, MIRU-VNTR could help reduce the impact of a false positive diagnosis of TB.

## Methods

### Samples

Clinical specimens were processed according to standard methods and grown in Lowenstein-Jensen slants and in MGIT (Becton Dickinson, Sparks, Md) liquid media.

### Molecular analysis

For IS6110-RFLP we followed the standard procedures [15], and for MIRU-VNTR we applied the 12-loci set [8], trying to apply the simplest and fastest MIRU format and also attempting to obtain a result by directly amplifying a crude extract of the culture (after boiling and sonicating for ten and five minutes, respectively). MIRU-VNTR products were separated by electrophoresis at 45 V for 17 h 30 min, using MS8 2% agarose gels (Pronadisa, Madrid, Spain). Fragment sizes were calculated with the ChemiDoc system (BioRad, CA, USA) and the Diversity database (BioRad), using a 100-bp ladder (Invitrogen, CA, USA) as a molecular weight marker. The number of repeats in each locus was calculated by applying the corresponding conversion tables (P. Supply, personal communication)

Molecular patterns were analyzed using Bionumerics 4.6 (Applied Maths, Sint-Martens Laten, Belgium). Results were interpreted as false positivity if both the potential source and the cross-contamination alert had identical MIRU-VNTR and IS6110 RFLP and the converse for true positivity.

## Competing interests

The author(s) declare that they have no competing interests.

## Authors' contributions

AM performed all the VNTR-MIRU assays, analyzed the results and was involved in the first version of the MS. MH performed the IS6110-RFLP assays, analyzed the results and was involved in the first version of the MS. MML performed all the microbiological procedures and identified the cross-contamination alerts. He has been involved in the compilation of all microbiological and clinical data essential for the analysis of the alerts RFF was involved in the analysis of all the clinical and epidemiological data required for the analysis of the alerts. EB revised critically the final version of the MS. DGV* designed the study, supervised all experimental work, analyzed the results, corrected and produced the final version of the MS. INDAL-TB group was involved in the compilation and analysis of microbiological, clinical and epidemiological data relevant for the analysis of the cross-contamination alerts and critically reviewed the final version of the MS. All the authors read and approved the final MS.
